# Characterization of the circRNA Landscape in Interleukin-4 Induced Anti-Inflammatory Microglia

**DOI:** 10.3390/biomedicines11123239

**Published:** 2023-12-07

**Authors:** Chaoyi Li, Huakun Wang, Yu Tang, Junjiao Wu

**Affiliations:** 1 Department of Geriatrics, Xiangya Hospital, Central South University, Changsha 410008, China; lcy_lnsn@163.com (C.L.); whk4311579@outlook.com (H.W.); 2 National Clinical Research Center for Geriatric Disorders, Xiangya Hospital, Central South University, Changsha 410008, China; 3 Department of Rheumatology and Immunology, Xiangya Hospital, Central South University, Changsha 410008, China; 4 Provincial Clinical Research Center for Rheumatic and Immunologic Diseases, Xiangya Hospital, Central South University, Changsha 410008, China

**Keywords:** neurodegenerative disease, neuroinflammation, microglia, circRNA, circAdgre1

## Abstract

Microglia are resident innate immune cells that play an essential role in the development and surveillance of the central nervous system as well as the shared pathogenesis of neurodegenerative diseases. Microglia rapidly respond to multiple inflammatory stimuli and activate towards different phenotypes, such as pro-inflammatory and anti-inflammatory phenotypes. Cytokines, epigenetic and long non-coding RNA modulations have been shown to regulate microglial activation; however, the role of circRNAs in microglia-mediated neuroinflammation remains elusive. Here, we performed circRNA sequencing in IL-4-treated anti-inflammatory microglia and discovered 120 differentially expressed circRNAs. We systemically verified the identities of circRNAs by assays of PCR, RNase R treatment and fluorescent in situ hybridization (FISH), among others. We found that circAdgre1 promoted IL-4-induced anti-inflammatory responses and further conferred neuroprotective effects upon lipopolysaccharide (LPS) stimuli. Taken together, our results show that circRNAs might be possible therapeutic targets for microglia-mediated neuroinflammation and neurodegenerative diseases.

## 1. Clinical Perspectives

The potential role of circRNAs in microglia-mediated neuroinflammation, the shared pathogenesis of neurodegenera-tive diseases, remains elusive. We performed circRNA sequencing in IL-4-treated anti-inflammatory microglia and discovered potential differentially expressed circRNAs, which were later experimentally validated. In particular, we found that circAdgre1 promoted anti-inflammatory responses and further conferred neuroprotective effects upon LPS stimuli. Our results thus show that circRNAs might be potential therapeutic targets for microglia-mediated neuroinflammation and neurodegenerative diseases.

## 2. Introduction

The prevalence of neurodegenerative diseases is growing rapidly worldwide. Patients with neurodegenerative diseases, such as Alzheimer’s disease (AD), Parkinson’s disease (PD) and amyotrophic lateral sclerosis (ALS), often have symptoms such as memory loss, dyskinesia, muscle weakness, atrophy, spasticity, chorea, dystonia and so forth [1,2]. Along with the disease development, patients might gradually lose their ability to live independently, which gravely dampens their life quality. However, effective treatment strategies are still limited [3]. The deposition of aberrant proteins, such as β-amyloid (Aβ) and tau in AD [4,5], α-synuclein in PD [6], SOD1 and TDP-43 in ALS [7], HTT polyQ in HD [8] and polyG in NIID [9], among others, is recognized as the shared pathology of multiple neurodegenerative diseases.

Microglia are innate immune cells in the central nervous system (CNS) and play versatile roles in the maintenance of milieu homeostasis and neural development by regulating synaptic pruning [10]. Upon stimulation, microglia can be rapidly activated into various phenotypes with alterations in morphology, phagocytic capacity, or the release of inflammatory factors [11,12], which could be simply classified as pro-inflammatory and anti-inflammatory phenotypes during neuroinflammation [13,14]. Microglial activation manifests a dynamic alteration during the development of AD; activation in an early stage can engulf the aberrant deposited proteins and cell debris, thus alleviating the disease pathogenesis. However, activation might develop into chronic dysfunction along with the disease progression due to persistent stimuli, including aberrant protein depositions, skewing into pro-inflammatory phenotypes that may exacerbate the disease [15]. Therefore, regulating microglial activation might be a promising therapeutic strategy of neurodegenerative diseases.

Mounting studies have shown that circRNAs participate in the pathogenesis of neurodegenerative diseases [16]. With the advancement of circRNA sequencing technology, differentially expressed circRNAs have been discovered in neurodegenerative diseases, such as AD [17,18,19,20,21]. CircRNAs are basically derived from the back splicing of pre-mRNAs, which are covalent single-strained loops without a poly A tail at the 3′ end or a cap structure at the 5′ end; the circular structure makes circRNAs more resistant to exonucleolytic enzymes [22]. Most circRNAs are derived from protein-coding exons, while a small number of circRNAs are derived from introns and the intergenic portion of the genome [23]. CircRNAs can regulate gene expression by modulating the transcription of their parental genes [24,25]. Also, circRNAs can serve as miRNA sponges or bind with RNA-binding proteins (RBPs) to regulate the expression of downstream molecules [26,27,28,29], and, interestingly, a variety of circRNAs can even synthesize short peptides or proteins using intrinsic IRES elements [30,31,32,33,34]. Notably, circRNAs are highly conserved among species and primarily accumulate in the postmitotic cells, especially inside the CNS [28,35,36,37], suggesting that circRNAs might play an important role in the growth and development of the brain as well as the pathogenesis of neurological diseases.

In this study, we aimed to characterize the circRNA landscape in anti-inflammatory microglia and discover potential anti-inflammatory circRNAs. To this end, we carried out circRNA sequencing in IL-4-treated anti-inflammatory microglia and identified 120 differentially expressed circRNAs. The identities of the circRNAs were further verified by PCR, RNase R treatment and fluorescent in situ hybridization (FISH), among others. We revealed that circAdgre1 could promote IL-4-induced anti-inflammatory responses, as assessed by the overexpression and knockdown using the CRISPR/Cas13d system, and further conferred its neuroprotective effects upon lipopolysaccharide (LPS) stimuli. This study thus suggests that circRNAs might be possible therapeutic targets for mediating neuroinflammation during the development of neurodegenerative diseases.

## 3. Materials and Methods

### 3.1. Cell Culture

BV2 cells from the murine microglia cell line were routinely cultured in a complete culture medium and incubated at 37 °C with 5% CO_2_. The complete medium contained Minimum Essential Medium (MEM, Procell, Wuhan, China) supplemented with 1% NEAA, 10% heat-inactivated FBS (Biological Industries, Beit-Haemek, Israel) and 1% penicillin/streptomycin. Also, 293T and N2A cells were cultured in DMEM medium (Procell) supplemented with 10% FBS and 1% PS.

### 3.2. Sample Preparation for circRNA Sequencing

We cultured BV2 cells with serum-free MEM containing IL-4 (20 ng/mL, Peprotech, Cranbury, NJ, USA); after 8 h of incubation, the cells were digested with trypsin-EDTA (0.25%, Gibco, Norristown, PA, USA) for 2 min, followed by adding an equal volume of the complete medium to halt the digestion. The mixture was centrifuged at 5000× *g* for 2 min. The supernatant was then removed while cell pellets were washed by DPBS 3 times. One mL of TRIzol solution (Invitrogen, Carlsbad, CA, USA) was added respectively to each tube of cell pellets and, finally, the samples were sent for circRNA sequencing (Ribo Biotech, Guangzhou, China).

### 3.3. Library Construction and Sequencing

Total RNAs were isolated from microglia according to the manufacturer’s manual. The RNA purity was assessed by ND-1000 Nanodrop requiring A260/280 ≥ 1.8 and A260/A230 ≥ 2.0. The RNA integrity (RIN) was evaluated by Agilent 2200 TapeStation (Agilent Technologies, Santa Clara, CA, USA) requiring RIN ≥ 7.0. Briefly, rRNAs were removed from total RNAs using the Epicentre Ribo-Zero rRNA Removal Kit (Illumina, Hayward, CA, USA), followed by treatment with RNase R (Epicentre, San Francisco, CA, USA) and fragmentation into approximately 200 bp. Subsequently, the purified RNA fragments were subjected to first-strand and second-strand cDNA synthesis, followed by adaptor ligation and enrichment with a low cycle, according to the instructions of the NEBNext Ultra RNA Library Prep Kit for Illumina (NEB, Ipswich, MA, USA). The purified library products were then evaluated using Agilent 2200 TapeStation and Qubit 2.0 (Life Technologies, Carlsbad, CA, USA) and then sequenced on HiSeq 3000 in 2 × 150 bp mode.

### 3.4. Pre-Processing of Sequencing Reads

Raw reads were treated with Trimmomatic tools (v0.36) to remove adapters. Following reads quality control, reads were scanned with a 4-base wide sliding window, cutting when the average quality per base dropped below 15, i.e., drop reads that were less than 35% of initiation read length. Reads quality was inspected using the FastQC software (Version 0.11.9) before outputting statistical results (Supplementary Materials).

### 3.5. Identification and Quantification of circRNAs

Two algorithms, CIRI2 and CIRCexplorer2, were used to detect circRNAs. Reads were mapped to human reference genome GRCh37/hg19 (http://genome.ucsc.edu/ accessed on 20 October 2019) by BWA-MEM or TopHat, respectively. CIRI2 detects paired chiastic clipping (PCC) signals from the mapping information of reads by local alignment with BWA-MEM and combines with systematic filtering steps to remove potential false positives. CIRCexplorer2 uses TopHat and TopHat-Fusion alignment output to detect circRNAs.

If a circRNA could be detected by both methods, it was considered an identified circRNA (Supplementary Materials). Back-spliced junction reads identified in CIRI2 were combined and scaled to RPM (reads per million mapped reads, BWA-MEM mapping) to quantify every circRNA.

### 3.6. circRNA Amplification and Sanger Sequencing

Divergent circRNA primers were designed respectively with the help of circPrimer, a software for annotating circRNAs and determining the specificity of circRNA primers [38]. circRNAs were amplified by PCR with EasyTaq DNA Polymerase (TransGen Biotech, Beijing, China) according to the manufacturer’s instructions. PCR products were separated by 1% agarose gel electrophoresis and, later, DNA bands with correct sizes were further cut out respectively for sanger sequencing.

### 3.7. Real-Time qPCR

Total RNAs were extracted from cultured cells using AG RNAex Pro Reagent (Accurate Biotech, Guangzhou, China) and, later, cDNA synthesis reactions were performed using 2 µg of RNA from each sample with the First Strand cDNA Synthesis Kit (ThermoFisher, Waltham, MA, USA) and random hexamer primers. Real-time qPCR was performed using the SYBR Green Premix Pro Taq HS qPCR Kit (Accurate Biotech) and run on Quant Studio DX (ABI System Inc., Anaheim, CA, USA). The expression levels were normalized to the housekeeping genes GAPDH or HPRT by the 2^−ΔΔCt^ method. Primer sequences are listed in Supplementary Table S1.

### 3.8. Prediction of circRNA Secondary Structures

The prediction of circRNA secondary structures was performed with the RNAfold web server [39] (http://rna.tbi.univie.ac.at/cgi-bin/RNAWebSuite/RNAfold.cgi accessed on 4 March 2021) and also displayed with the mountain plot.

### 3.9. RNase R Treatment

In total, 5 μg of total RNA was mixed with 2 μL 10 × RNase R reaction buffer, 0.25 μL RNase R (Geneseed Biotech, Guangzhou, China) and an appropriate amount of H_2_O to a total volume of 20 μL. The mixture was incubated in a 37 °C water bath for 10 min and then a 70 °C water bath for 10 min to inactive the enzyme. The products were run by agarose gel electrophoresis. This assay was used to detect the resistance of circRNAs to RNase R exonuclease digestion.

### 3.10. FISH

The FISH Kit (Ribo Biotech) was used to examine the subcellular localization of circRNAs according to the manufacturer’s instructions. The Cy3-labeled circAdgre1 probe was specifically designed to span the BSJ site (Ribo Biotech). Briefly, BV2 cells were seeded on a 24-well plate till 60~70% confluency was reached. Cells were washed with DPBS for 5 min and fixed with 4% PFA at room temperature (RT) for 10 min. Cells were treated with a permeabilization solution for 5 min and washed with DPBS 3 times. Each well of cells was incubated with 200 µL of a pre-hybridization buffer at 37 °C for 30 min. Later on, the buffer was discarded and 2.5 µL of the mixture was incubated with a 20 µM circAdgre1 probe and 100 µL of a hybridization buffer at 37 °C overnight. Cells were washed with hybridization wash buffer I (4 × SSC, 0.1% Tween-200) at 42 °C 3 times for intervals of 5 min. Later on, cells were washed with hybridization wash buffer II (2 × SSC) at 42 °C once and with hybridization wash buffer III (1 × SSC) at RT once. Cells were lastly counter-stained with DAPI for 5 min and mounted with anti-fade PVA for further observation. Images were taken using a Zeiss LSM 710 confocal microscope.

### 3.11. Cloning and Constructs

For overexpression, the full length of circAdgre1 was cloned into EcoRI/BamHI sites of the pLCDH-CiR vector (Geneseed Biotech, Guangzhou, China). The amplified primers were as follows:

Forward: CGGAATTCTGAAATATGCTATCTTACAGGTGTGAATGAGTG TCAAGATACTACC; reverse: CGGGATCCTCAAGAAAAAATATATTCACCT TCACAAATGGAGCCATTC

For knockdown, the guide RNA (gRNA) sequences for circAdgre1 were designed with the help of Cas13design, an online webserver (https://cas13design.nygenome.org/ accessed on 10 March 2021). The following gCircAdgre1 oligos were synthesized and annealed into duplexes:

gCircAdgre1-1—forward: AAACCACACCTTCACAAATGGAGCCAT; reverse: AAAAATGGCTCCATTTGTGAAGGTGTG.

gCircAdgre1-2—forward: AAACTCACACCTTCACAAATGGAGCCA; reverse: AAAATGGCTCCATTTGTGAAGGTGTGA.

pLentiRNACRISPR-hU6-DR-EFS-RfxCas13d (Addgene 138147) was used as the knockdown vector and was digested with BsmBI (NEB) at 37 °C for 30 min for use. Fifty ng digested vector and 200 ng annealed oligos were mixed respectively with T4 DNA ligase (Thermofisher, Waltham, MA, USA) according to the manufacturer’s instructions. The reaction was incubated at room temperature for 30 min and transformed into Trans5α chemically competent cells (TransGen Biotech). Generated plasmids were amplified from each single colony and verified by Sanger sequencing before use.

### 3.12. Virus Package and Infection

Each of the lentiviral plasmids was co-transfected with packaging plasmids (psPAX2 and pMD2.G) into 293T cells for lentivirus production. The culture media containing lentiviruses were collected respectively at 48 h and 72 h after transfection. The collected virus was then filtered through 0.45-μm syringe filters and stored at 4 °C before use. BV2 cells were seeded at 60~70% confluency in 12-well plates. The next day, cells were infected with a virus supplemented with 8 μg/mL polybrene. The medium was replaced 24 h later for further use.

### 3.13. Conditioned Medium, ELISA and Neural Survival

Cultured BV2 cells were treated with LPS (100 ng/mL, Sigma, Granville, Australia) for 8 h and the culture media were then harvested and filtered through 0.45 mm filters for use. The concentrations of TNF-α and IL-6 in the culture media were determined using the ELISA kits (ABclonal) according to the manufacturer’s instructions. For the cytotoxicity assay, N2A cells were seeded at a density of 5 × 10^3^ cells/well on a 96-well plate overnight before being changed into conditioned medium, and, the next day, were cultured with the conditioned medium for 24 h. CCK-8 assay was performed to assess cell viability. Briefly, 20 μL of CCK-8 solution (Beyotime, Shanghai, China) was added to the wells and the plate was incubated for 2 h at 37 °C. After that, ODs at 450 nm were measured to determine the cell viability.

### 3.14. Data Analysis

GraphPad Prism (Version 8.0.1) software was utilized for statistical analysis and graphing. Student’s *t*-test was used for statistical analysis, with a 95% confidence level considered significant regarding differences between groups. A *p*-value of less than 0.05 indicated statistical significance.

## 4. Results

### 4.1. Overview of circRNA Profiles in Anti-Inflammatory Microglia

**Figure 1:**
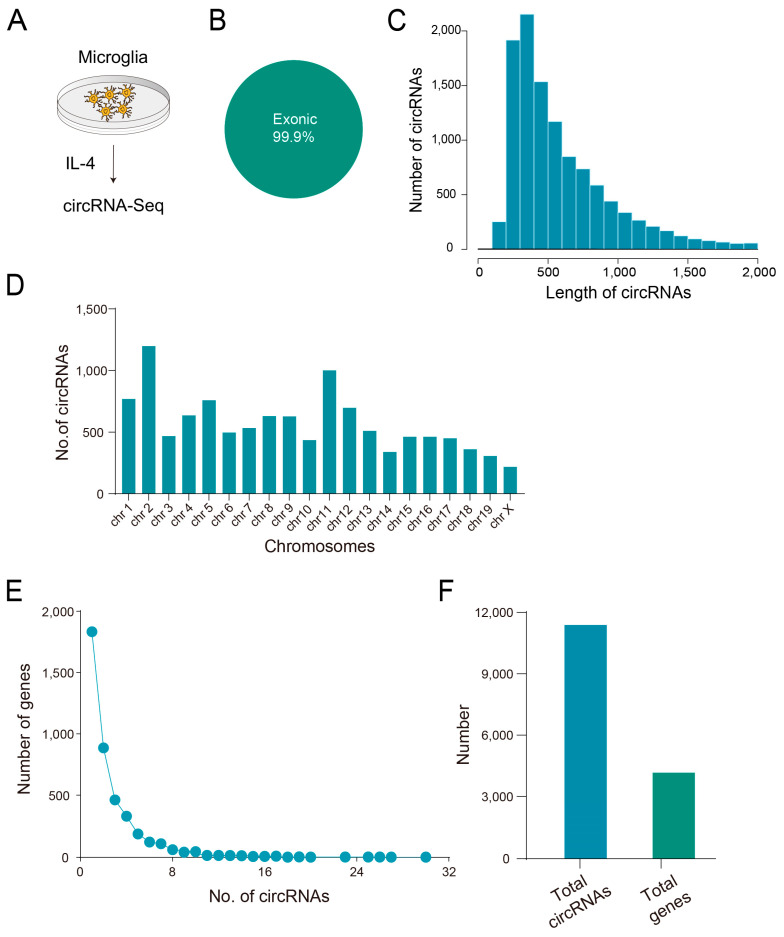
Overview of circRNA profiles in anti-inflammatory microglia: (**A**) Microglia treatment strategy. (**B**) CircRNAs derived from exons account for 99.9%. (**C**) CircRNA length distribution. (**D**) Schematic diagram of circRNA distribution on chromosomes. (**E**,**F**) Correspondence between circRNAs and parental genes.

To detect the differential expression of circRNA in anti-inflammatory microglia, we treated BV2 cells with anti-inflammatory factor IL-4 for 8 h and later collected cells for circRNA sequencing (Figure 1A). After depleting rRNAs and linear RNAs, total RNAs were used to prepare libraries for circRNA-seq. circRNAs were identified by both CIRI2 and CIRCexplorer2 algorithms.

In our study, a circRNA that could be detected by both methods was considered an identified circRNA. We then discovered over 4000 circRNAs, most of which are novel, and further predicted the functional elements (exons, introns, 5′UTR, 3′UTR or intervals) and their derived genes by annotating the junction sequence of circRNAs. The annotation results showed that 99.9% of circRNAs were derived from the exons (‘exonic’) (Figure 1B). The predicted length of circRNAs ranged in length from 200 bp to 1000 bp, with the majority population of circRNAs being 200~400 nt (Figure 1C). The circos plot shows that the host genes of circRNAs were distributed widely on the chromatin, especially on chromosomes 2 and 11, suggesting that circRNAs are of significantly large quantification in microglia (Figure 1D). Since host genes generally possessed multiple transcripts, each host gene might have produced a diversity of circRNAs due to the various types of RNA splicing, and the maximum number of produced circRNA in a single host gene was as great as 30 (Figure 1E,F).

### 4.2. Identification of Differentially Expressed circRNA Profiles

**Figure 2:**
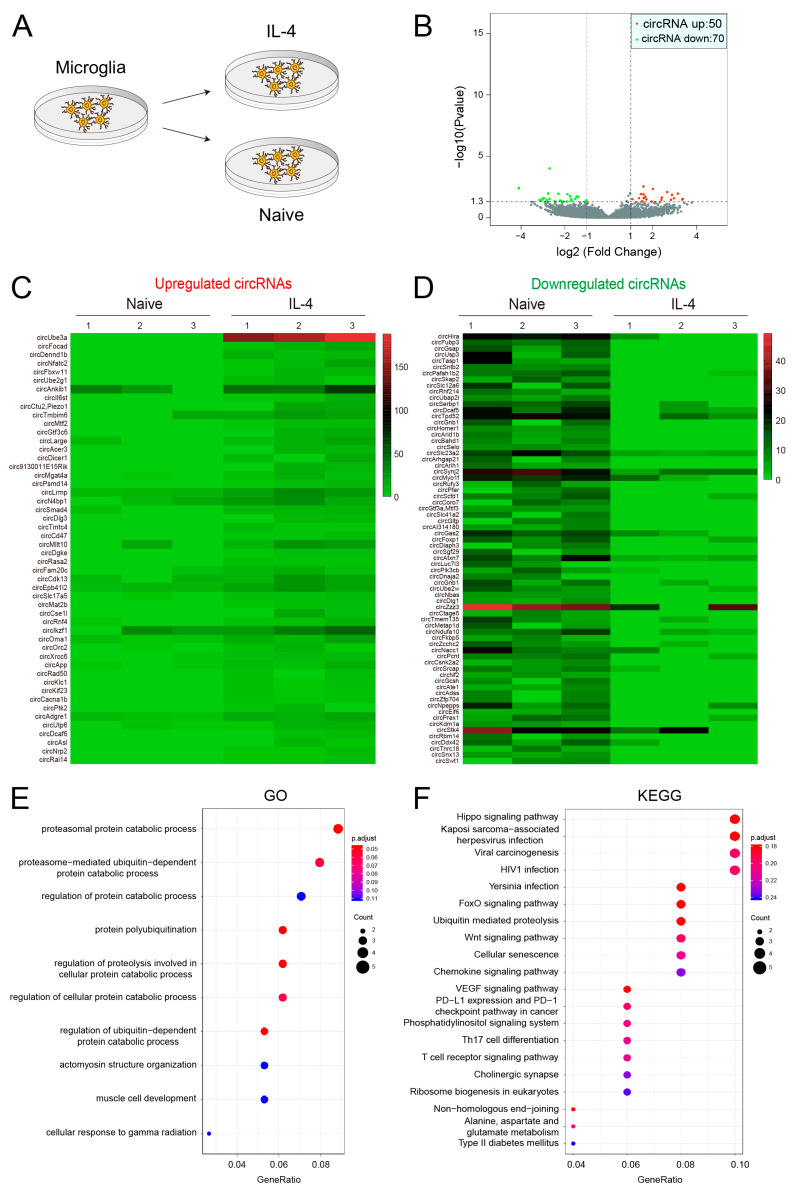
Differential expression of circRNAs in IL-4-treated microglia: (**A**) Sample preparation for circRNA sequencing. (**B**) Volcano plot of 120 differentially expressed circRNAs. (**C**,**D**) Clustering heat map of upregulated and downregulated circRNAs. (**E**,**F**) The GO and KEGG analyses of differentially expressed circRNAs.

We utilized DESeq to identify the differential expression of circRNAs and obtained 50 upregulated circRNAs and 70 downregulated circRNAs in IL-4 treated cells compared to those in the untreated group (Figure 2A,B). Those differentially expressed circRNAs were also displayed in the heat map, respectively (Figure 2C,D). Considering that the circRNAs were generated by mRNA splicing and some circRNAs functioned by regulating their parental mRNAs, we could imagine changes in potential biological pathways in which the circRNAs may have been involved. To assess the biological roles of differentially expressed circRNAs, we then performed gene ontology (GO) analysis and Kyoto encyclopedia of genes and genomes (KEGG) analysis. GO analysis revealed that the overlapping circRNAs were significantly enriched in the proteasomal protein catabolic process, proteasome-mediated ubiquitin-dependent protein catabolic process, regulation of the protein catabolic process protein polyubiquitination, and so on (Figure 2E). In the KEGG analysis, four pathways including the hippo signaling pathway, kaposi sarcoma-associated herpesvirus infection, viral carcinogenesis and human immunodeficiency virus 1 infection were considered the most relative pathways (Figure 2F).

To further explore the identities of those differentially expressed circRNAs, we compared the circRNA profiles in our study with those in previous studies that carried out circRNA profiling in neurodegenerative diseases, such as Dube et al.’s results [17]. In their study, they used Mount Sinai Brain Bank (MSBB) and Knight Alzheimer Disease Research Center (ADRC) data to compare the expression of circRNAs with diverse risk factors during the development of AD. We thus aligned our differential circRNAs with theirs (Supplementary Figure S1). Based on clinical dementia ratings, we discovered three shared circRNAs in “MSBB” and “DOWN”: circPREX1, circTASP1 and circATE1; one shared circRNA in “ADRC”, “MSBB” and “DOWN”: circHOMER1; and one shared circRNA in “MSBB” and “UP”: circPTK2. Based on neuropathological AD traits, we discovered two shared circRNAs in “MSBB” and “UP”, circPTK2 and circANKIB1, and three shared circRNAs in “MSBB” and “DOWN”, circHOMER1, circPREX1 and circATE1 (Supplementary Figure S1).

### 4.3. Validation of the Differentially Expressed circRNAs

**Figure 3:**
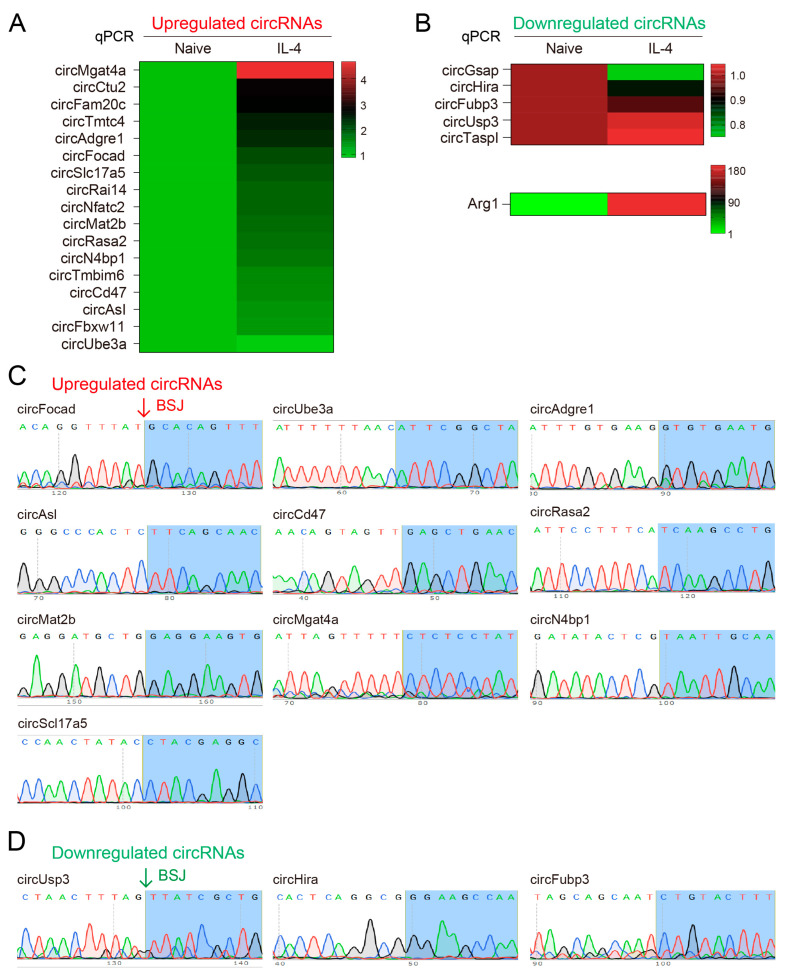
Validation of differentially expressed circRNAs: (**A**,**B**) Validation of differentially expressed circRNAs by real-time qPCR. The Arg1 expression was set as positive control. (**C**,**D**) Sanger sequencing of amplified circRNAs. All circRNAs were verified by spanning BSJ sites.

As covalent single-strand non-coding RNAs, circRNAs could be identified by PCR with divergent primers crossing the back-splicing junction (BSJ) sites. To validate the circRNA sequencing data, the expressions of upregulated and downregulated circRNAs were experimentally verified, respectively. Specifically, we designed divergent primers for over 40 upregulated circRNAs and 5 typically downregulated circRNAs, with the help of circPrimer, to specifically amplify circRNAs (Figure 3A,B). By performing real-time qPCR, we verified 14 circRNAs upregulated >1.5 fold upon IL-4 treatment, whereas the fold changes of downregulated circRNAs were milder (Figure 3A,B). To our surprise, the expression of circUbe3a, which was predicted to be the most increased circRNA in the sequencing data, showed mild changes in the qPCR verification (Figure 3A). Another interesting circRNA, circIL6st, whose host gene is strongly associated with immune regulation, was hardly detectable after using more than three pairs of different divergent primers.

Later on, PCR products were confirmed by agarose gel electrophoresis and were further sanger sequenced to verify the back-splicing junctions (BSJs). The results showed that the amplified products, from either upregulated or downregulated circRNAs, indeed crossed the BSJs, which demonstrated their circRNA identities (Figure 3C,D).

### 4.4. Characterization of the circAdgre1 Identity

**Figure 4:**
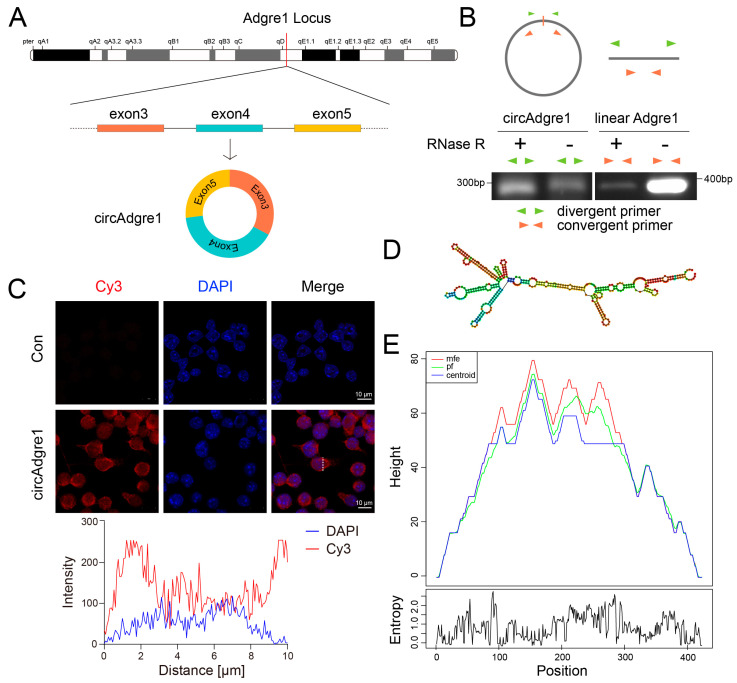
Characterization of the circAdgre1 identity: (**A**) The formation of circAdgre1 by back-splicing from the host gene. (**B**) Schematic diagram of nucleic acid electrophoresis and circular formation of total RNA with RNase R treatment. (**C**) Subcellular localization of circAdgre1 was assessed by FISH and shown to be cytoplasmic. (**D**,**E**) The secondary structure of circAdgre1 was predicted by RNAfold web server and displayed as a mountain plot, showing several key loops that might serve as miRNA sponges or RBP binding sites.

After assessing the biological functions of verified circRNAs and their host genes, we recognized circAdgre1 as the most interesting since Adgre1, also known as F4/80, is mainly expressed in immune cells such as macrophages and is widely studied in immune responses. We then compared circAdgre1 with its cognate linear forms and discovered that circAdgre1 was derived from the back splicing of *Adgre1* exon 3, exon 4 and exon 5 (Figure 4A).

Since circRNAs are covalent single-stranded circular RNAs without a 3′ poly-A tail or a 5′ cap, they are more resistant to exonuclease digestion and are more stable than linear RNAs. In our case, upon RNase R treatment, circAdgre1 amplified by divergent primers became resistant to degradation and even enriched to some extent, whereas its cognate linear Adgre1 mRNA amplified by convergent primers was extremely sensitive (Figure 4B). We further performed FISH analysis and showed that circAdgre1 is mainly located in the cytoplasm, which is basically in line with the notion that exonic circRNAs are cytoplasmic (Figure 4C).

The secondary structure of circRNA plays an essential role in RNA interactions [40]. For example, circKcnt2 could inhibit the activation of group 3 innate lymphoid cells to facilitate colitis resolution, while its loop HR5 was required for the association between circKcnt2 and the BATF promoter [41]. Therefore, we also predicted the secondary structure of circAdgre1 with the RNAfold web server and mountain plot, showing several key loops that might serve as miRNA sponges or RBP binding sites (Figure 4D,E). As a parallel control, we also characterized another upregulated circRNA, circFocad, and obtained similar features (Supplementary Figure S2).

As circRNAs could act as miRNA sponges, we further predicted the potential miRNA binding sites to circAdgre1 via miRanda, RNAhybrid and TargetScan algorithms and selected their intersection data for analysis. We constructed the circRNA–miRNA network and showed almost 100 miRNAs predicted to bind with circAdgre1, including a variety of immune-related miRNAs (Supplementary Figure S3; Supplementary Table S2).

### 4.5. circAdgre1 Overexpression Promotes Anti-Inflammatory Responses

To further explore the potential effects of circAdgre1 in inflammatory responses, we employed both circRNA overexpression and knockdown vectors to assess the role of circAdgre1 under gain- and loss-of-function conditions. Notably, we applied the newly developed CRISPR/RfxCas13d system to knockdown circRNA expression, which was shown to perform better than short hairpin RNAs (shRNAs) or siRNAs in terms of their specificity in lowering the expression of circRNAs but not their cognate linear mRNAs [42,43,44].

**Figure 5:**
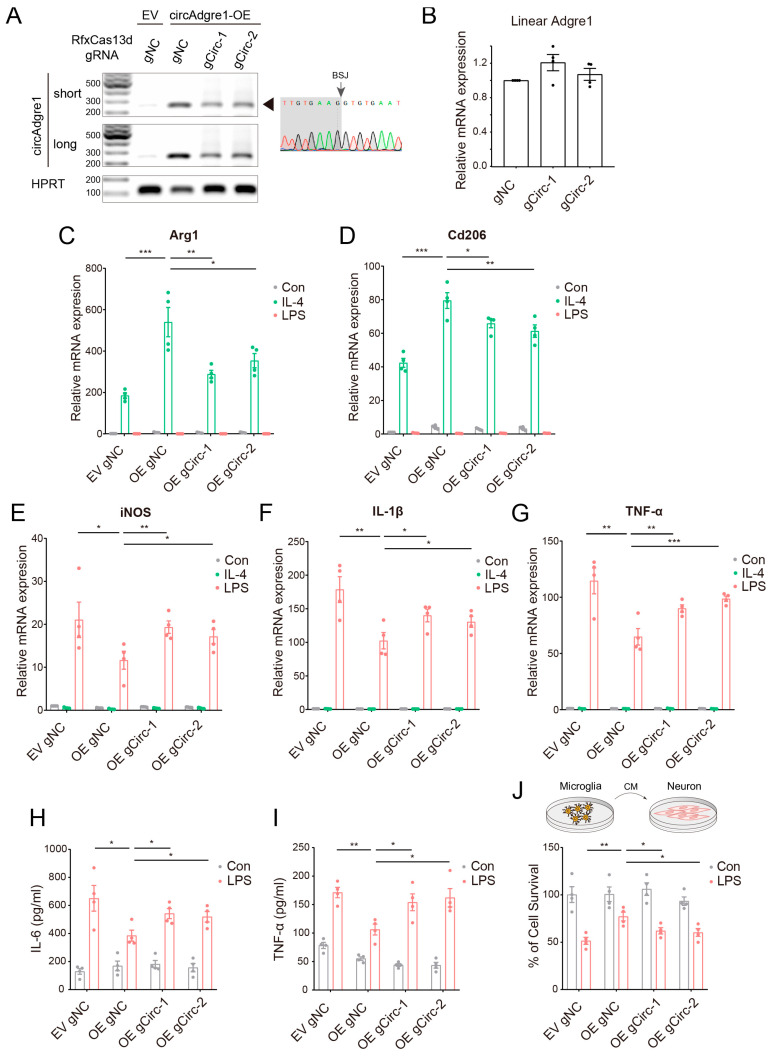
circAdgre1 overexpression promotes anti-inflammatory responses: (**A**) Validation of overexpression and knockdown efficiency of circAdgre1. The PCR product was sent for sanger sequencing and confirmed that the overexpressed circAdgre1 indeed crossed the BSJ site. (**B**) The expression level of cognate linear Adgre1 mRNA was detected by real-time qPCR. (**C**,**D**) The expression levels of anti-inflammatory markers including Arg1 and Cd206 were assessed by real-time qPCR. (**E**–**G**) The expression levels of pro-inflammatory markers including iNOS, IL-1β and TNF-α were assessed by real-time qPCR. (**H**,**I**) The concentrations of TNF-α and IL-6 in the culture medium were measured respectively using ELISA. (**J**) The conditioned medium of microglia cells was harvested for culturing neuron cells, and cell viability was detected by CCK-8 assay. * *p* < 0.05, ** *p* < 0.01, *** *p* < 0.001.

Our results showed that the full length of circAdgre1 was successfully overexpressed, which was also confirmed by circular RNAs through sequencing the BSJ site (Figure 5A). Both RfxCas13d-gRNAs indeed reduced circAdgre1 expression without compromising the level of linear Adgre1 mRNA (Figure 5A,B). We then infected microglia cells with overexpressed and knockdown viruses, followed by LPS or IL-4 treatment. We examined the expression of inflammatory factors and found that circAdgre1 overexpression significantly elevated the expression of anti-inflammatory markers including Arg1 and Cd206 after IL-4 treatment, which was inhibited upon circAdgre1 knockdown (Figure 5C,D). In the case of LPS stimuli, the increased levels of pro-inflammatory factors, such as iNOS, IL-1β and TNF-α, were greatly suppressed but could be partially restored by circAdgre1 knockdown (Figure 5E–G). Moreover, ELISA measurements of the culture medium showed significantly higher levels of TNF-α and IL-6 upon LPS treatment, which were suppressed by circAdgre1 overexpression (Figure 5H,I). These results suggest that circAdgre1 overexpression promotes anti-inflammatory microglial responses.

To evaluate the effects of circAdgre1 on neuron cytotoxicity, we treated microglia cells and collected the conditioned medium, which was later used for culturing N2A neurons. We performed a CCK-8 assay to measure cell viability and found that overexpressing circAdgre1 in LPS-treated cells alleviated their toxic effects on neurons; the ratio of surviving neurons was elevated from 51.37 ± 2.46% to 77.03 ± 3.08% (Figure 5J). Taken together, those results conferred circAdgre1 as an anti-inflammatory and neuroprotective circRNA and also suggested that circRNAs might be possible therapeutic targets for microglia-mediated neuroinflammation and neurodegenerative diseases.

## 5. Discussion

In our study, we showed that the number of circRNAs is significantly higher than that of cognate linear RNAs, albeit with much lower expression abundance. Note that the expression of circRNAs is tissue-specific [17]; for example, in the brain, circRNA levels in the olfactory bulb, prefrontal cortex, hippocampus and cerebellum are higher than those in other brain regions [45]. circRNAs can also gradually accumulate in synapses with aging [46], which may be caused by the dysfunction of alternative splicing during senescence [47]. The above studies have suggested that circRNAs are actively involved in the physiology of the CNS.

Along with the progress of sequencing technology, the differential expression of circRNAs has gained increasing attention in the field of neurodegenerative diseases. For instance, a broad range of differentially expressed circRNAs was detected in the brains of AD patients [17,18,19,20,21], the levels of which were significantly correlated with the neuropathological changes and clinical severity of AD [17]. Among the 37 circRNAs identified in the parietal cortex, circHOMER1 showed a significant correlation with AD, while the increased expression of circCDR1as greatly aggravated dementia [17]. Interestingly, the expression of circRNA was altered in the early stage of the disease, which is of great significance for early diagnosis. Dynamic circRNA expression levels could also be detected in the peripheral blood mononuclear cells (PBMCs) of PD patients [48]. Once circRNA expression profiles are ensured, this non-invasive detection method might render the diagnosis of PD easier. For example, circSLC8A1, which is highly expressed in the substantia nigra (SN) of PD patients, has seven binding sites for miR-128 and displays a high affinity with AGO2 proteins [49]. circSLC8A1 could aggravate intracellular oxidative stress, thereby exacerbating disease development [49]. Thus, circRNAs are highly related to the pathogenesis of neurodegenerative diseases and are expected to serve as biomarkers and therapeutic targets.

In this study, circRNA sequencing was performed on IL-4-induced anti-inflammatory microglia and more than 4000 circRNAs were detected, of which 120 circRNAs displayed differential expression. Differentially expressed circRNAs might be involved in the hippo signaling pathway observed in the KEGG analysis. The hippo signaling pathway is associated with pathophysiological states such as cell survival, proliferation and apoptosis [50]. Notably, hippo signaling is also linked to the development of neurodegenerative diseases [51]. As a critical component of the hippo signaling pathway, MST1 can induce neuronal apoptosis upon oxidative stress [52], while the deposition of Aβ can promote MST1-mediated FOXO3 phosphorylation [53]. In the hippocampus of transgenic APP^swe^/PS1^dE9^ AD mice, the expressions of hippo signaling-related genes, such as c-ABL, p-MST and p-YAP, were increased [54]. Alterations in hippo signaling can also be measured in HD. The YAP subunit YAP^deltaC^ continuously increases during the pathogenesis of HD and acts as a YAP-TEAD antagonist to regulate neuronal apoptosis [55]. Dysfunction in hippo signaling was also detected in the post-mortems of HD patients, with increased levels of p-MST and p-YAP, whereas YAP levels in neuronal nuclei were significantly decreased [56]. Similarly, in patients with ALS, the hippo pathway was activated and YAP levels in the nuclei of the cerebral cortex were greatly reduced [57].

Adgre1, also known as F4/80, is mainly expressed in the cell membranes of macrophages and is a marker of macrophages. Macrophages mainly include Kupffer cells in the liver, red plasma macrophages in the spleen, microglia in the CNS and Langerhans cells in the intestine and skin [58,59,60]. Adgre1 is involved in cell adhesion, the regulation of cellular communication between immune cells, and the growth and development of regulatory T cells. Interestingly, in whole-brain circRNA sequencing, the expression levels of circAdgre1 in white matter microglia were higher than those in gray matter microglia [61]. We discovered that circAdgre1 overexpression produced anti-inflammatory and neuroprotective effects, suggesting that circRNAs could vitally regulate immune responses. However, the molecular mechanisms of circAdgre1 in anti-inflammation merit further investigation. circAdgre1 might either serve as miRNA sponges or directly dampen the production of its cognate Adgre1 mRNA with the inclusion of circle-forming exons.

There are over 100 miRNAs predicted to combine with circAdgre1. circRNAs and miRNAs form a competitive endogenous RNA (ceRNA) network. For example, circSmek1 levels are increased during neuropathic pain, along with decreased miR-216a-5p [62]. Either the knockdown of circSmek1 or the overexpression of miR-216a-5p can reduce the production of TNF-α, IL-1β and IL-6 in the spinal cord, whereas they promote microglia polarization towards anti-inflammatory phenotypes [62]. Furthermore, circSmek1 induces TXNIP expression by competitively adsorbing miR-216a-5p, forming a mode by which circSmek1 regulates the miR-216a-5p/TXNIP axis to promote the pro-inflammatory response of neuropathic pain [62]. Specific miRNAs that may bind with circAdgre1 await deeper investigation.

## 6. Challenges and Limitations of This Study

The largest challenge was that murine circRNAs were not comprehensively indexed in most recognized circRNA databases, such as circBase [63], circBank [64] and circInteractosome [65], which brought extra difficulty to the annotation and functional dissection of candidate circRNAs. Moreover, cell-specific circRNA databases such as TSCD [66] and circSC [67] did not involve any microglia data. Notably, the intrinsic challenge came from the much lower expression abundance of circRNAs compared to their cognate linear host genes, which can also be observed in Figure 4B and Supplementary Figure S2B. Due to the low abundance, we did not obtain a clear result when we directly knockdowned circAdgre1 in BV2 cells, but, instead, we overexpressed circAdgre1 and meanwhile knocked it down by RfxCas13d-gRNAs. It remains unknown whether, upon the presence of strong stimuli, changes in the majority of circRNAs are actively executed functions or just derived from the byproducts of massive linear RNA expression echoing the requirement of rapid responses to immune stimuli.

As we only plotted the circRNA atlas for murine microglia cells, the profiles of differentially expressed circRNAs in the specimens of patients or in vivo mice models remain elusive. Specifically, the BV2 microglia line contains oncogenes that render them different from primary microglia in some ways, such as proliferation, adhesion and the variance of morphologies. Moreover, as microglia are highly heterogeneous during neural development, disease pathogenesis and the aging process [12], the circRNA landscape in this study is rather simplified under a defined context or microglial state. Whether circAdgre1 plays a dynamic role in other contexts or at different pathogenesis timepoints, as well as the roles of more potential circRNAs in human samples or in vivo models, will need further studies to address this.

## Data Availability

Data are available upon request from the corresponding author.

## Supplementary Materials

The following supporting information can be downloaded at https://www.mdpi.com/article/10.3390/biomedicines11123239/s1; Figure S1: The comparison of differentially expressed circRNAs in different studies. The differentially expressed circRNAs in this study were compared to those in the study by Dube et al. 2019. (A) For clinical dementia rating, we found 3 shared circRNAs in “MSBB” and “DOWN”: circPREX1, circTASP1, circATE1; 1 shared circRNA in “ADRC”, “MSBB” and “DOWN”: circHOMER1; 1 shared circRNA in “MSBB” and “UP”: circPTK2. (B) For neuropathological AD traits, we found 2 shared circRNAs in “MSBB” and “UP”: circPTK2, circANKIB1; 3 shared circRNAs in “MSBB” and “DOWN”: circHOMER1, circPREX1, circATE1; Figure S2: Characterization of the circFocad identity. (A) The formation of circFocad by back splicing from the host gene. (B) Schematic diagram of nucleic acid electrophoresis and circular formation of total RNA with RNase R treatment. (C,D) The secondary structure of circFocad was predicted by RNAfold web server and displayed as mountain plot; Figure S3: The potential miRNA binding sites of circAdgre1. The circAdgre1-miRNA network was in silico established. Almost 100 miRNA binding sites on circAdgre1 were predicted, reminiscent of the possibility of circAdgre1 serving as miRNA sponges; Table S1: Sequences of divergent primers for circRNA detection; Table S2: The list of miRNAs predicted to bind with circAdgre1.
