# Supplementary material for: Characterization of the circRNA Landscape in Interleukin-4 Induced Anti-Inflammatory Microglia
Source: Biomedicines. 2023 Dec 7;11(12):3239. doi: 10.3390/biomedicines11123239 (PMC10740700; doi:10.3390/biomedicines11123239)
Supplement: Supplementary file 1 [file biomedicines-11-03239-s001.zip › biomedicines-2658582-supplementary.pdf]

Supplementary Materials

The statistics of the circRNA-seq mapping

1. rRNA reads filtering

In the process of library construction, there will be a small amount of ribosomal RNA (rRNA) fragments remaining, resulting in the existence of some rRNA reads in the data. To avoid affecting the subsequent analysis of the data, we remove the known rRNA sequences by comparing them with the rRNA sequences in the RNAcentral database, and obtain the data with the rRNA reads removed for the subsequent analysis. The statistics are shown in the table below, and the effective_reads will be used in the next step of genome alignment.

Type	Ctrl-1	Ctrl-2	Ctrl-3	IL4-1	IL4-2	IL4-3
Clean_reads	146,071,972 (100%)	118,941,766 (100%)	158,856,688 (100%)	126,193,342 (100%)	136,237,890 (100%)	149,802,546 (100%)
rRNA_reads	10,285,600 (7.04%)	8,855,422 (7.45%)	9,414,366 (5.93%)	17,221,910 (13.65%)	15,028,334 (11.03%)	11,212,610 (7.48%)
The_effective_reads	135,786,372 (92.96%)	110,086,344 (92.55%)	149,442,322 (94.07%)	108,971,432 (86.35%)	121,209,556 (88.97%)	138,589,936 (92.52%)

Note:

[1] Clean_reads: number of reads in Clean Data after quality control and percentage of Clean_reads;

[2] rRNA_reads: number of rRNA reads and percentage of Clean_reads;

[3]The_effective_reads: number of effective reads for circRNA analysis and percentage of Clean_reads.

2. Genome alignment

We used both CIRI2 and CIRCexplorer2 algorithms for circRNA identification. Reads were mapped to human reference genome GRCh37/hg19 by BWA-MEM or TopHat, respectively. Since the results of CIRI2 algorithm were preferred for the subsequent calculation of circRNA expression, the results of genome alignment for CIRI2 are listed in the table below.

Sample	Total Reads	Mapped Reads	Mapped Reads%
Ctrl-2	110,086,344	109,794,762	99.74%
Ctrl-1	135,786,372	135,390,490	99.71%
Ctrl-3	149,442,322	149,093,721	99.77%
IL4-1	108,971,432	108,485,871	99.55%
IL4-2	121,209,556	120,845,719	99.70%
IL4-3	138,589,936	138,234,069	99.74%

3. CircRNA identification

We used CIRI2 for circRNA identification. CIRI2 uses the result file of BWA-MEM alignment to the genome as the input file, and predicts the position information before and after the formation of circRNA for each sample individually, and further identifies the candidate circRNA sequences. The results of each sample are shown in the table below.

Sample	CIRI2 circRNAs	CIRCexplorer2 circRNAs	Overlapped circRNAs
Ctrl-1	8,664	8,635	4,856
Ctrl-2	7,192	7,496	4,159
Ctrl-3	8,289	9,516	5,030
IL4-1	5,564	5,529	3,036
IL4-2	7,848	8,702	4,764
IL4-3	7,359	7,720	4,195

Supplementary Tables

Supplementary Table S1. Sequences of divergent primers for circRNA detection

Name	Divergent Primer_F	Divergent Primer_R
mmu_circUbe3a	TCACCCTGATGTCACCGAAT	GCTGCTAACTTGATCTGAACGT
mmu_circFocad	TGCAAGCACTGAGAGAAGGA	CCGTCTGGTTTGTGGATTGG
mmu_circDennd1b	AGTAAGCAGCGATTTGGGTTC	AGGTAAAGTGCTGTCCAACTTG
mmu_circNfatc2	CATTCCCATCTGCAGCATCC	GGCTGGTTCGAGGTGACATT
mmu_circFbxw11	CAGGTGGAATTTGTGGAGCA	GCACTTTTCTTTGGGGACTCA
mmu_circUbe2g1	AAAGATTATCCCCTCCGGCC	AACCTGCTGAAAAGCCTTCC
mmu_circAnkib1	ATGAAGGACTAAGGCCCCAG	AGTGCTTTGCGGAATTTGGT
mmu_circCtu2	TGCCCATGGTCATCTTCAGT	AGCACGAATGTACTTGGGGA
mmu_circTmbim6	ATGTATGTTTGTGGCGGCAG	TGTCGAGGGAGTTATGTGGG
mmu_circMtf2	ACAGAGCTGCTTCATCATATTTG	AGCCATCTGACCATCTAGCT
mmu_circGtf3c6	GAACGAGGGAATTGACACGG	ACATAGCTGTCCACTTGCATG
mmu_circLarge	ATTCCTGGCTGCCTCTTTGA	CCCAGCATCCTCTCAGAAGT
mmu_circAcer3	CAGGTCATGTATGGAATGTTGGT	AGCTCTTTGTCTTGAAACACTCA
mmu_circDicer1	TGCTGTGAGTTTCCAATGAGC	GCTCTCGAAATGCTGACTCT
mmu_circ9130011E15Rik	GCACCAGAGCTTTGACAACC	TGTTGTGCTTGGCCAAAAGT
mmu_circMgat4a	TCCTGAAGAGAAGCTGGACTG	GCGGAACTGCTGGACAATG
mmu_circPsmd14	GACTTCTTAGACTTGGAGGAGGT	TCCAAGTCTTGCTAATTCTGGC
mmu_circLrmp	TCTGGTGACCTTGACTGCAA	TGCTCTCCGAGGATGAAGTC
mmu_circN4bp1	ACCAGGACGAGCAGATTTGA	GAGGTCAGCACACGTATCCT
mmu_circSmad4	GGACTGCACCATACACACCT	AAGACATCTGGTGAGCAGGA
mmu_circDlg3	ACCTGAGGAATGCTACCCAC	CTGCGATACTGAAGCCAAGG
mmu_circTmtc4	TCAGTACTGTTTGGTGGCCT	AGGACTTGTGGCTGGTGTTA
mmu_circCd47	GGCCTTCAACACTGACCAAG	CATTACGGACGATGCAAGGG
mmu_circMllt10	AACATGTGCTCAGTTTGCCG	TCAGGGTTAGGCAAAGTGTC
mmu_circDgke	CCACTGTGCTCAAATCCAGG	AGCGTGAAAATTGAGAGCCA
mmu_circRasa2	GCAGTTACAGCAGGAAGTCC	CAATCGCGCATTTTGTTGGG
mmu_circFam20c	AGTGGCTGGTAGGATGATCA	TGGAAGGCAGCGATCTCC
mmu_circCdk13	ACGGGAGAAATGGTAGCCTT	CTTCGGGATTTGGCAGATCG
mmu_circEpb41l2	GATGGCACGGAATACAGCTG	ACCCTTTTCTTCCTCTGGCT
mmu_circSlc17a5	AGCCGCTGACTATTTAAGGGT	CCACCTTCTGCGAAGAAAGC
mmu_circMat2b	GGGCTGCTGTGTTGAGAATT	CACCAGTAATGAGAACCCGC
mmu_circCse1l	GCCTCCTGAATCAGTTGACC	AACCTTGGTGATCGTTTGCC
mmu_circApp	GACACACATGGCCAGAGTTG	TTGGCTTTCTGGAAATGGGC
mmu_circRad50	GCCGTGATGAAATGCTTGGG	CTCGCTGCTTTGAGGACTTT
mmu_circKlc1	AAAACCCTGGGCAGAGATCA	TTCTCGGACTCCACAGCATT
mmu_circKif23	TCGGATGATCGTGTGTGTGA	GTTGGTTCTTTCGCTCCCAG
mmu_circCacna1b	TCCCCAACAGCACAGATACA	CCTCGAAGCAAAAGATCCCG
mmu_circPtk2	GTGCCAGATGTGAGAGCTCT	AACTCAGAAGGCAGCAGTGA

mmu_circAdgre1	TGCGCAGATGTTGATGAGTG	TGGACAGGAAGCCTCGTTTA
mmu_circUtp6	TCCAGGTGCTCATTGACTCA	TCCATTTCCCATTTGGCTGC
mmu_circDcaf6	AATCAACTGTAGGCGTGCTG	CACAGCCATCATGCACATTTAA
mmu_circAsl	GCAGAAGCGGATCAATGTCT	GTCCTGTGTGTAGCTTCCCT
mmu_circNrp2	GGGGAAAACTGCAGCTTTGA	GTGGCATGTGGAGCTTGTTT
mmu_circRai14	TCTCTCGGACACAATGCCTT	ATCTTGGGCAGTCACATCCA
mmu_circHira	CAGCACTGTGTTTGGTTCCA	AGAATCCTGCCCTTGTCCTC
mmu_circFubp3	CGAACGGGTGTGAAAATGGT	AAGGCCCCTAACTGGTTACC
mmu_circGsap	AGGATTTTGGATGCTGCTAG	TGCAGGTGTCTCAGTAGGTG
mmu_circUsp3	AGGAAGGCGAACATACCACA	ACCACAAAATCGTCACAGCG
mmu_circTaspl	CAGGCTGCTCTTTATGGATGT	AGTGCCTGAGTCATTTTCCTG

Supplementary Table S2. The list of miRNAs predicted to bind with circAdgre1

miR-125a-3p	miR-1969	miR-6993-5p
miR-140-3p	miR-664-3p	miR-7004-3p
miR-145a-5p	miR-3057-5p	miR-7008-5p
miR-152-3p	miR-3057-3p	miR-7012-5p
miR-153-5p	miR-3077-5p	miR-7037-5p
miR-181a-5p	miR-3078-5p	miR-7044-5p
miR-182-5p	miR-3082-5p	miR-7056-5p
miR-188-3p	miR-3087-5p	miR-7063-5p
miR-191-3p	miR-3092-3p	miR-7074-5p
miR-194-1-3p	miR-3097-5p	miR-7080-5p
miR-143-5p	miR-3962	miR-7086-5p
miR-96-5p	miR-5107-5p	miR-7087-5p
miR-326-5p	miR-5108	miR-7088-5p
miR-320-3p	miR-1231-5p	miR-7222-5p
miR-181b-5p	miR-5626-3p	miR-7230-3p
miR-380-5p	miR-6347	miR-7234-3p
miR-468-3p	miR-145b	miR-7242-3p
miR-758-5p	miR-6396	miR-7651-5p
miR-671-5p	miR-6402	miR-7662-3p
miR-1298-5p	miR-3473e	miR-7668-3p
miR-344d-2-5p	miR-6905-5p	miR-7669-3p
miR-681	miR-6915-3p	miR-7677-3p
miR-505-5p	miR-6917-5p	miR-6715-5p
miR-181d-5p	miR-6925-5p	miR-7688-5p
miR-574-3p	miR-6926-5p	miR-1291
miR-873a-3p	miR-6932-5p	miR-8100
miR-1197-5p	miR-6937-5p	miR-497b
miR-1902	miR-6938-5p	miR-3154
miR-1894-5p	miR-6947-5p	miR-9b-3p
miR-1930-3p	miR-6957-5p	miR-12188-5p
miR-1953	miR-6958-5p	miR-12196-3p
miR-1955-5p	miR-6958-3p	

Supplementary Figures

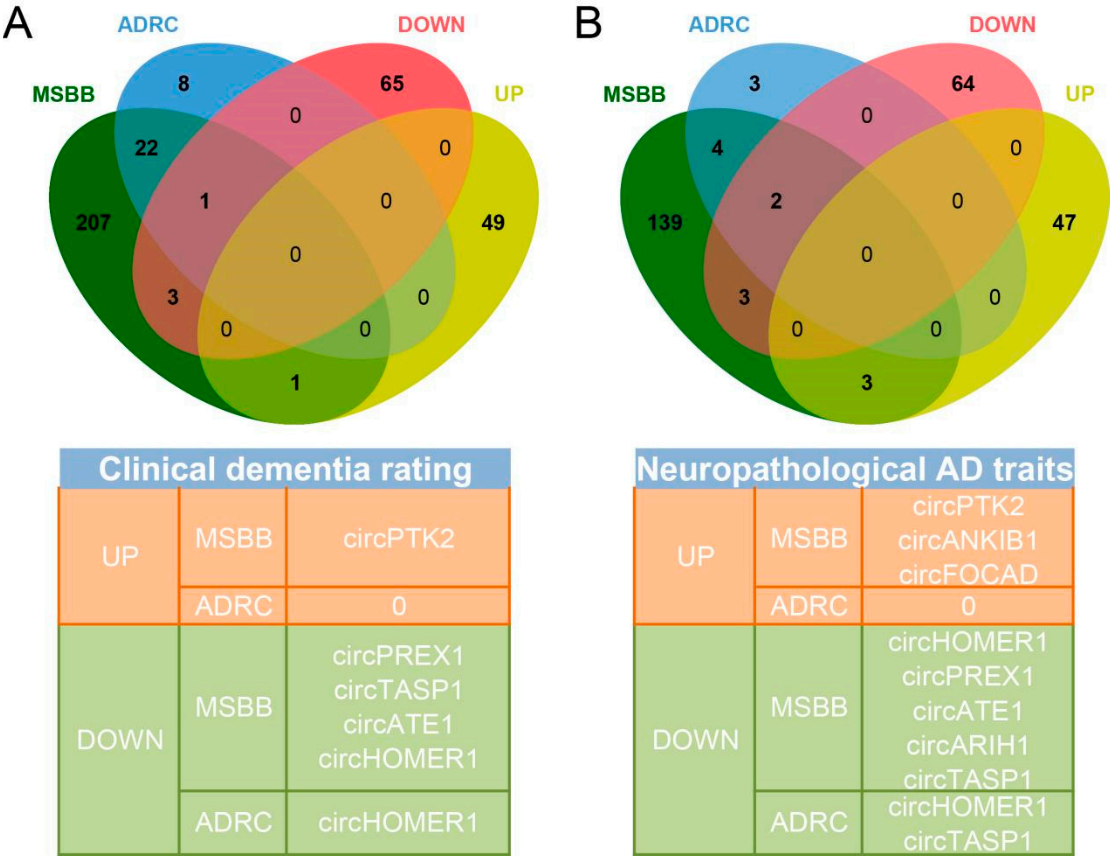

Supplementary Figure S1 The comparison of differentially expressed circRNAs in different studies

The differentially expressed circRNAs in this study were compared to those in the study by *Dube et al. 2019*. (A) For clinical dementia rating, we found 3 shared circRNAs in "MSBB" and "DOWN": circPREX1, circTASP1, circATE1; 1 shared circRNA in "ADRC", "MSBB" and "DOWN": circHOMER1; 1 shared circRNA in "MSBB" and "UP": circPTK2. (B) For neuropathological AD traits, we found 2 shared circRNAs in "MSBB" and "UP": circPTK2, circANKIB1; 3 shared circRNAs in "MSBB" and "DOWN": circHOMER1, circPREX1, circATE1.

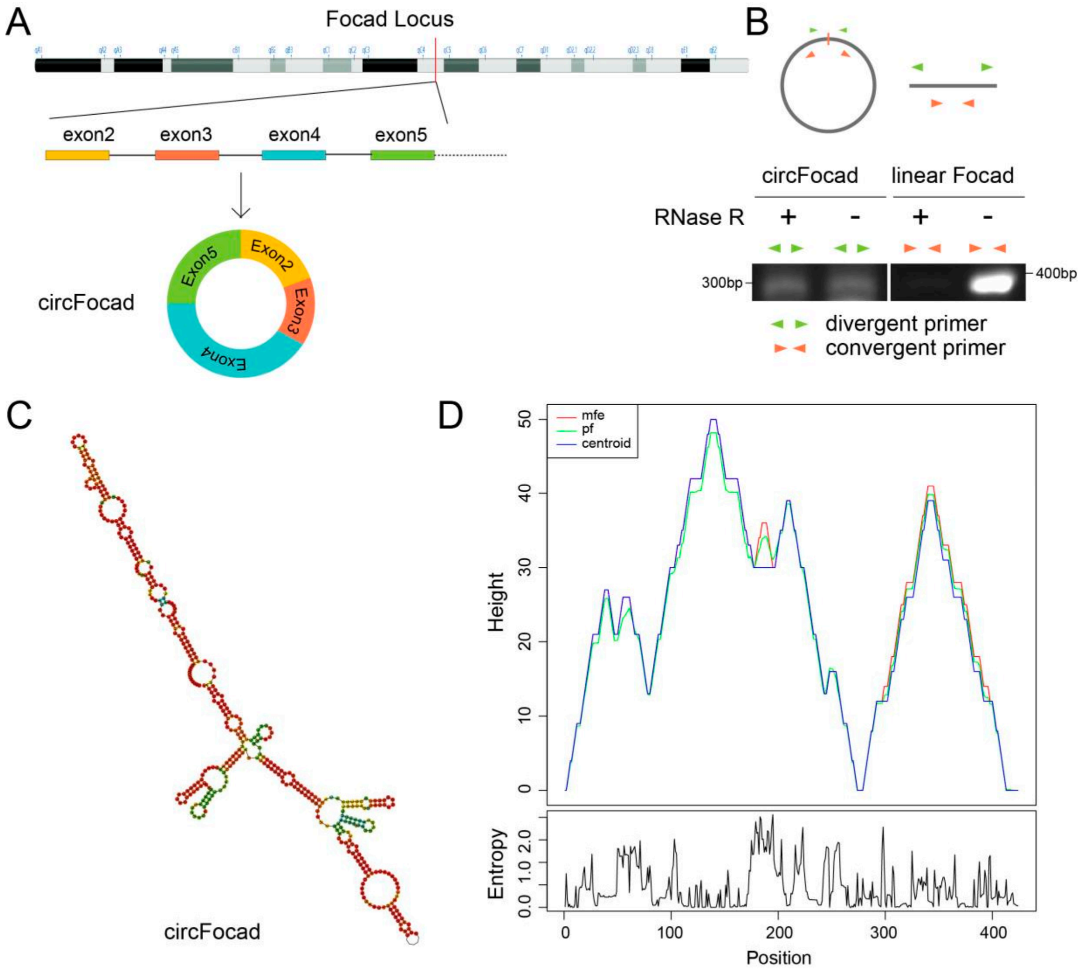

Supplementary Figure S2 Characterization of the circFocad identity

(A) The formation of circFocad by back splicing from the host gene. (B) Schematic diagram of nucleic acid electrophoresis and circular formation of total RNA with RNase R treatment. (C, D) The secondary structure of circFocad was predicted by RNAfold web server and displayed as mountain plot.

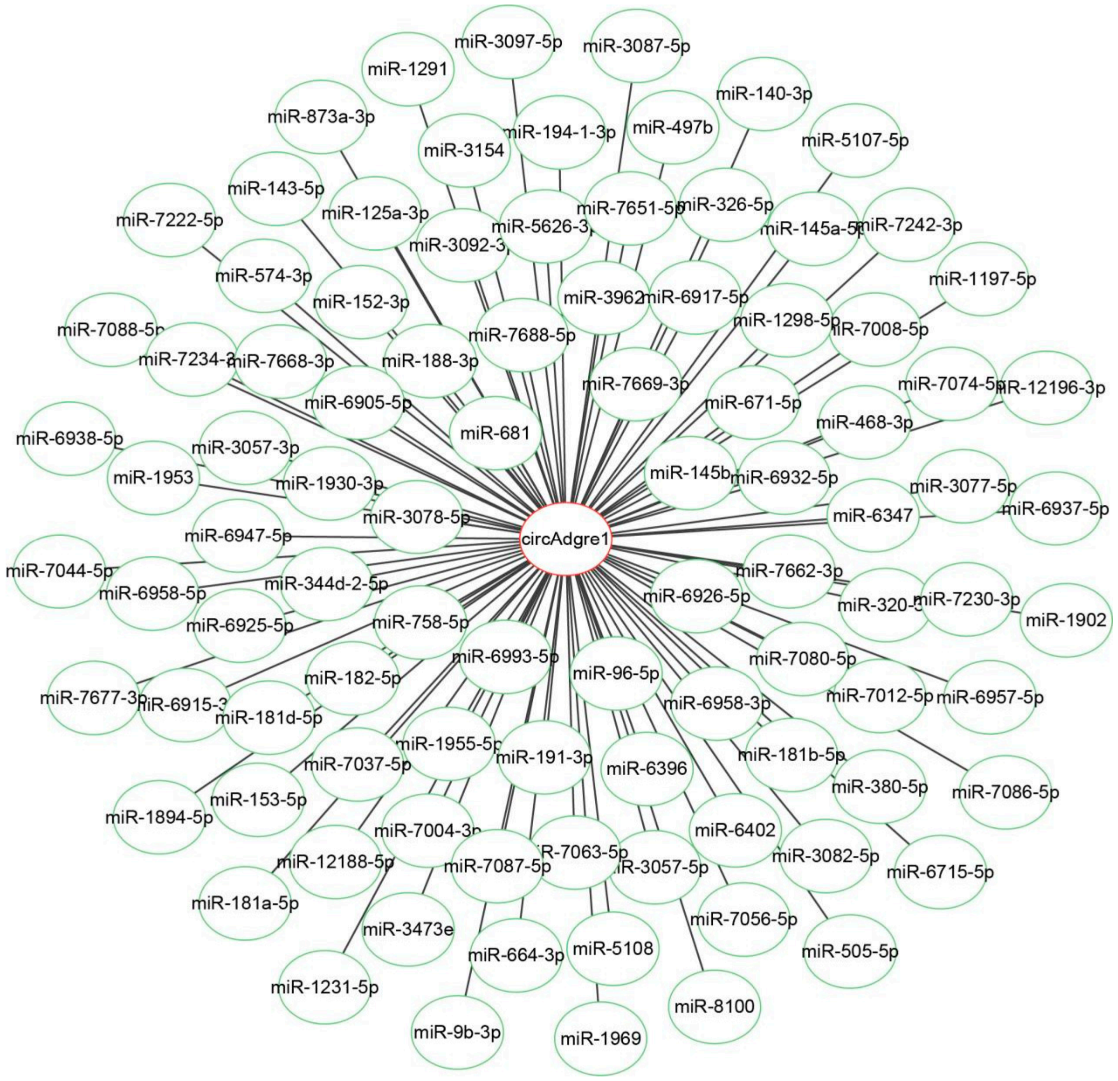

Supplementary Figure S3 The potential miRNA binding sites of circAdgre1

The circAdgre1-miRNA network was *in silico* established. Almost 100 miRNA binding sites on circAdgre1 were predicted, reminiscent of the possibility of circAdgre1 serving as miRNA sponges.
